# The relationship between fasting blood glucose, glycated hemoglobin, fructosamine, and depression in patients with type 2 diabetes mellitus in South Korea: a secondary analysis of the Korea National Health and Nutrition Examination Survey VIII-2 (2020) data

**DOI:** 10.7586/jkbns.25.083

**Published:** 2026-02-27

**Authors:** Jeesun Lee

**Affiliations:** Department of Nursing, Korea Nazarene University, Cheonan, Korea

**Keywords:** Diabetes mellitus, type 2, Fructosamine, Glycated hemoglobin, Depression

## Abstract

**Purpose:**

This study examined the associations between fasting blood glucose (FBG), glycated hemoglobin (HbA1c), fructosamine, and depression among adults with type 2 diabetes mellitus (T2DM) using nationally representative data from the Korea National Health and Nutrition Examination Survey (KNHANES).

**Methods:**

A descriptive cross-sectional study was conducted using secondary data from the second year (2020) of the eighth KNHANES. Adults diagnosed with T2DM were included. Depression was assessed using the Patient Health Questionnaire-9. Complex-sample analyses were applied to account for the survey design, and hierarchical multivariable logistic regression analyses were performed to evaluate associations between glycemic indices reflecting different temporal windows and depression.

**Results:**

Depression was not significantly associated with FBG. In contrast, depression prevalence was significantly higher among participants with normal fructosamine levels, whereas it was significantly lower among those with normal HbA1c levels. Younger age and smoking were associated with higher odds of depression, while cohabitation and employment were associated with lower odds.

**Conclusion:**

Among adults with T2DM, glycemic indices representing different time frames demonstrated distinct associations with depression. Long-term glycemic control, as indicated by HbA1c, was associated with lower odds of depression, whereas intermediate-term glycemic control, reflected by fructosamine, showed an opposite association. These findings underscore the importance of considering the temporal characteristics of glycemic markers when interpreting their relationships with depression and highlight the need for longitudinal studies to clarify underlying mechanisms.

## INTRODUCTION

### 1. Background

Diabetes accounted for 11.1% of diabetes-related deaths worldwide 2025 [1] and was the seventh leading cause of death in Korea in 2024, accounting for 21.7% of total deaths [2]. Type 2 diabetes mellitus (T2DM), which accounts for 90%~95% of all diabetes cases, is being diagnosed at increasingly younger ages. As a result, the duration of disease tends to be prolonged, increasing exposure to chronic hyperglycemia, hypoglycemia, and various diabetes-related complications [3,4]. The prevalence of depressive and anxiety symptoms in patients with diabetes is approximately two to four times higher than that in the general population [5]. Physiologically, diabetes-related metabolic dysregulation has been associated with alterations in brain function, while impaired peripheral glucose regulation may contribute to depressed mood [6]. Comorbid depression in patients with diabetes is known to negatively affect quality of life, increase functional disability and medical costs, and worsen overall disease prognosis [7].

More than 25% of patients with T2DM experience depression [8]. When T2DM and depression coexist, the risks of complications, noncommunicable diseases, and mortality are significantly higher [9]. Depression in patients with T2DM is closely associated with the long-term burden of disease self-management, including continuous blood glucose monitoring, adherence to dietary recommendations, regular physical activity, and pharmacological therapy. Previous studies have demonstrated that depression adversely affects diabetes management and treatment adherence, leading to poorer health outcomes in this population [8,10]. Therefore, identifying and appropriately managing depression is essential for improving treatment adherence and overall disease outcomes in patients with T2DM. In this context, objective and clinically applicable measures are required to assess and manage depression in individuals with diabetes [11].

Glycemic control is a core component of diabetes management, and glycated hemoglobin (HbA1c) is widely used as a standard indicator of long-term glycemic control. According to clinical guidelines, poor glycemic control in T2DM is defined by HbA1c levels of 6.5% or higher [2,12]. Common indicators of glycemic control in diabetes include fasting blood glucose (FBG), fructosamine, and HbA1c. FBG reflects plasma glucose levels during fasting and is commonly used for diabetes diagnosis and monitoring. Fructosamine reflects the glycation of serum proteins and indicates average glycemic status over the previous 2~3 weeks, influenced primarily by albumin turnover. It is particularly useful in clinical conditions such as hemoglobinopathies, anemia, or chronic kidney disease, where HbA1c measurements may be less reliable due to shortened red blood cell lifespan; in such cases, fructosamine may provide a more sensitive indicator of short-term glycemic changes [13]. HbA1c reflects average blood glucose levels over the preceding 2~3 months and is widely used for predicting diabetes-related complications and long-term outcomes [12].

Previous studies involving patients with T2DM have primarily focused on identifying determinants of glycemic control. Prior research has reported that younger age [14,15], longer disease duration [14,16-19], hypertriglyceridemia [14,20], larger waist circumference [14,21,22], and lower levels of resistance exercise [14,22-24] were associated with poor glycemic control. However, there remains a paucity of studies examining the relationship between glycemic control indicators and psychological factors, particularly depression. Studies investigating the association between HbA1c and depression in patients with T2DM have yielded inconsistent findings. Some research has reported that higher HbA1c levels were significantly associated with increased risk of depression [25-28], while other studies have demonstrated no significant association between HbA1c and depressive symptoms [29-31]. Limited research has examined the relationship between short-term glycemic indicators such as FBG and depression, with inconsistent findings [32]. Furthermore, fructosamine, which reflects glycemic control over 2~3 weeks, has been rarely investigated in relation to depression, particularly in Korean patients with T2DM, with no prior studies identified in this population. These inconsistent findings, combined with the limited research comparing different glycemic control indicators reflecting varying time periods (i.e., short-term, intermediate-term, and long-term), highlight a significant gap in understanding the complex relationship between glycemic control and depression in T2DM patients.

In summary, the existing literature reveals considerable variability in how different measures of glycemic control are associated with depression among individuals with T2DM, particularly regarding indicators that represent distinct timeframes of glucose exposure. Additionally, the reliance on HbA1c as the sole glycemic metric in much of the current research may restrict comprehensive understanding of the intricate connections between glucose regulation and depression.

Therefore, this study investigated the associations among FBG, fructosamine, HbA1c, and depression in patients with T2DM, analyzing data from the 2020 Korea National Health and Nutrition Examination Survey (KNHANES VIII-2)

### 2. Study aim

The primary objective of this study was to investigate factors related to depressive symptoms among individuals with T2DM, utilizing data from the 2020 KNHANES VIII-2. Specifically, this study examined differences in depression according to general characteristics, health behavior factors, anthropometric characteristics, and glycemic indices in patients with T2DM, and further determined factors independently associated with depression.

## METHODS

### 1. Study design

This study employed a cross-sectional design involving a secondary analysis of data from the 2020 KNHANES VIII-2. KNHANES is a nationally representative, standardized survey conducted by the Korea Disease Control and Prevention Agency to assess the health and nutritional status of the Korean population. Detailed information on the survey design, data collection procedures, and quality control has been described elsewhere in the official KNHANES reports [33].

### 2. Participants

**Figure 1.**
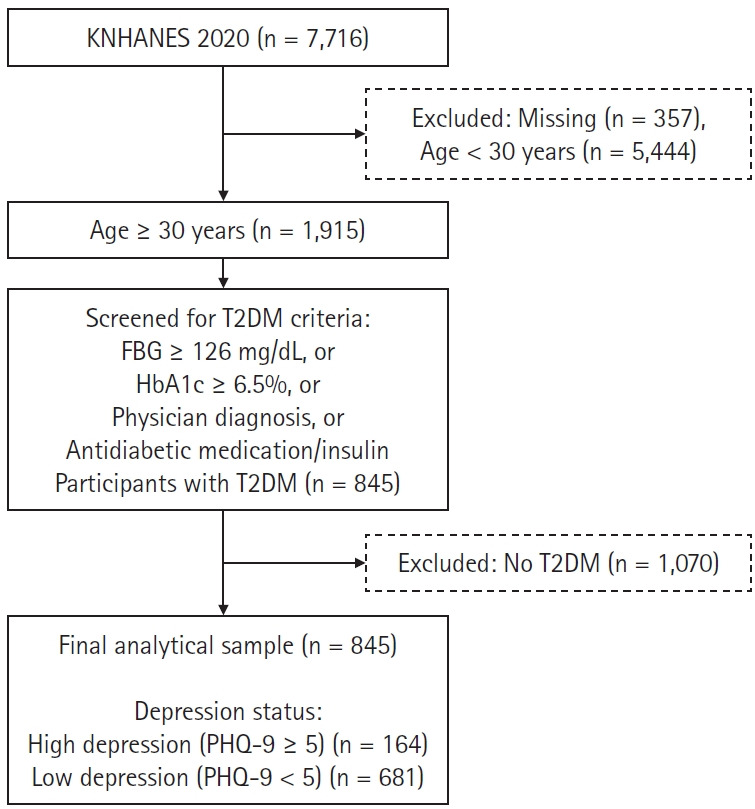
Flow diagram of study participant selection. KNHANES = Korea National Health and Nutrition Examination Survey; T2DM = Type 2 diabetes; FBG = Fasting blood glucose; HbA1c = Glycated hemoglobin; PHQ-9 = Patient Health Questionnaire-9.

Participants were adults aged 19 years or older who were identified as having T2DM. T2DM was defined as a FBG level of ≥ 126 mg/dL, a HbA1c level of ≥ 6.5%, current use of antidiabetic medication, or a previous diagnosis of diabetes by a physician [12,32]. In the 2020 KNHANES, a total of 7,716 participants were initially enrolled. Participants younger than 30 years (n = 5,444) and those with missing data on depressive symptoms or key glycemic indices (n = 357) were excluded, leaving 1,915 adults aged ≥ 30 years. Among these, 1,070 individuals who did not meet the diagnostic criteria for T2DM were further excluded. The final analytic sample thus comprised 845 participants (Figure 1).

### 3. Instruments

#### 1) General characteristics

General characteristics included sex, age, education level, cohabitation status, and employment status. Age was categorized as 30~49, 50~69, and ≥ 70 years. Education level was classified as elementary school or lower, middle school, high school, and college or higher. Cohabitation status was determined by household size (≥ 2 persons = “yes,” 1 person = “no”). Employment status was derived from economic activity survey items, with currently employed coded as “yes” and unemployed as “no.”

#### 2) Depression

Depressive symptoms were evaluated using the validated Korean translation of the Patient Health Questionnaire-9 (PHQ-9) [34]. Scores range from 0 to 27, with higher scores indicating greater severity. A cutoff score ≥5 was used to classify participants with depression [34].

#### 3) Anthropometric factors

Physical measurements comprised height (cm), weight (kg), body mass index (BMI, kg/m^2^), waist circumference (cm), systolic blood pressure (mmHg), and diastolic blood pressure (mmHg). BMI was calculated as weight divided by height squared and classified as underweight (< 18.5), normal (18.5~22.9), overweight (23.0~24.9), and obese (≥ 25). Waist circumference was classified as normal (< 90 cm for men , < 85 cm for women) or abnormal [35].

#### 4) Health behavior factors

Health behavior factors included smoking, alcohol consumption, and aerobic physical activity. Smoking status combined current use of cigarettes or heated tobacco (daily or occasional smoking = “smoker”; former or never = “non-smoker”). Alcohol consumption was assessed by binge drinking frequency (≥ 7 drinks for men, ≥ 5 for women) and classified as “no” (less than once per month or once per month) versus “yes” (once per week or more). Aerobic physical activity was classified as “yes” if participants reported ≥ 150 minutes of moderate-intensity activity, ≥ 75 minutes of vigorous activity, or an equivalent combination per week; otherwise, it was recorded as “no.”

#### 5) Glycemic factors

Glycemic indices included fasting glucose, HbA1c, and fructosamine. HbA1c was classified as good control (< 6.5%) and poor control (≥ 6.5%) according to the Korean Diabetes Association guideline [32]. Fructosamine levels were classified as good control (< 210 µmol/L) and poor control (≥ 210 µmol/L). Fasting glucose was classified as poor control at ≥ 126 mg/dL and good control at < 126 mg/dL, consistent with the diagnostic criteria applied for participant selection [12,32]. Laboratory procedures for blood sample collection and analysis followed standardized KNHANES protocols.

### 4. Data collection

The KNHANES extracts a representative sample of the Korean population aged ≥ 1 year. For this study, raw data were downloaded from the official KNHANES website (https://knhanes.kdca.go.kr) after submitting a formal request and receiving approval for data use [36].

### 5. Data analysis

Data were analyzed using SPSS version 25.0 (IBM Corp., Armonk, NY, USA), applying complex sample design to account for stratification, clustering, and weighting. Missing data were retained as valid categories in the complex sample analysis, in accordance with the KNHANES analytic guidelines [34], to avoid underestimation of standard errors (SEs). A significance level of *p* < .050 was applied. Descriptive statistics were presented as unweighted frequencies (n) and weighted percentages (%) for categorical variables, and as weighted means and SEs for continuous variables. Differences in depression according to sociodemographic, health behavior, anthropometric, and glycemic variables were tested using the chi-square test for categorical variables and the complex sample general linear model (t-test or analysis of variance) for continuous variables. Multivariable logistic regression analyses under the complex sample design were conducted to identify factors associated with depression.

### 6. Ethical considerations

Ethical clearance for the KNHANES VIII-2 was granted by the Korea Disease Control and Prevention Agency’s Institutional Review Board (IRB), and informed consent was secured from all subjects. The dataset used in this study was publicly available and fully de-identified.

For this study, IRB approval was obtained from the Ewha Womans University, with a waiver of written informed consent for secondary data analysis (IRB No. ewha-202205-0017-01).

## RESULTS

### 1. General characteristics and health behavior factors of the participants

The general characteristics and health behavior factors of the participants are presented in Table 1. Among the total 845 participants, 681 were classified as the non-depressed and 164 as depressed. Men accounted for 51.5% (n = 435) and women 48.5% (n = 410). The largest proportion of participants was aged 50~69 years (55.3%, n = 467). Regarding education level, 32% (n = 270) had completed elementary school or less. Most participants lived with others (79.4%, n = 671). About half were employed (49.7%, n = 420), while 50.3% (n = 425) were not employed.

With respect to health-related factors, 20% (n = 169) were current smokers. Binge drinking, defined as consuming ≥ 7 drinks for men or ≥ 5 drinks for women on a single occasion regardless of type, was reported as “none” by the majority of participants (82.4%, n = 696). Aerobic physical activity was practiced by 36.3% (n = 307). Regarding BMI, 50.9% (n = 430) were classified as obese. For glycemic indicators, 59.5% (n = 503) had FBG ≥ 120 mg/dL, 77.4% (n = 654) had fructosamine ≥ 210 µmol/L, and 73.7% (n = 623) had HbA1c ≥ 6.5%.

### 2. Differences in depression according to general characteristics and health behavior factors

Differences in depression levels according to general and health-related characteristics are shown in Table 1. Significant differences were observed for age (F = 17.60, *p* = .002), smoking status (χ^2^ = 13.90, *p* = .004), weight (t = 2.49, *p* = .013), and waist circumference (t = 3.05, *p* = .002), while no significant differences were found for the other variables.

### 3. Associations between depression and glycemic indicators

The associations between depression and glycemic indicators were examined using hierarchical multivariable logistic regression analyses, with results summarized in Table 2. To assess the incremental effects of conceptually distinct blocks of variables, predictors were entered sequentially based on their theoretical relevance and prior literature on depression in patients with T2DM. Model 1 included glycemic indicators only. Model 2 additionally adjusted for age, cohabitation status, which have been identified as important sociodemographic covariates associated with depression regardless of their statistical significance in univariable analyses. Model 3 further adjusted for smoking status, the only health behavior variable that showed a significant association with depression in univariable analyses and remained conceptually relevant after adjustment.

In Model 1, the risk of depression was 7.15 times higher in the normal fructosamine group (odds ratio [OR ] = 7.15, 95% confidence interval [CI ]: 1.99~25.68) and significantly lower in the normal HbA1c group (OR = 0.20, 95% CI: 0.06~0.67).

In Model 2, the risk of depression remained higher in the normal fructosamine group (OR = 6.65, 95% CI: 1.83~24.15) and lower in the normal HbA1c group (OR = 0.22, 95% CI: 0.06~0.76). Younger participants had a 3.75-fold higher risk of depression (OR = 3.75, 95% CI: 2.08~6.76). Those living with others had a lower risk compared to those living alone (OR=0.60, 95% CI: 0.37~0.98), and those employed had a lower risk than those unemployed (OR = 0.60, 95% CI: 0.38~0.94).

Although weight and waist circumference showed significant associations with depression in univariable analyses, these variables were not retained in the hierarchical models to avoid multicollinearity and over-adjustment, as they are closely correlated with other anthropometric indicators and may act as intermediates rather than independent confounders.

In Model 3, the risk of depression remained higher in the normal fructosamine group (OR = 6.77, 95% CI: 1.79~25.56) and lower in the normal HbA1c group (OR = 0.22, 95% CI: 0.06~0.79). Younger age was associated with a higher risk (OR = 3.07, 95% CI: 1.58~5.99). Living with others showed a lower odds of depression compared to living alone; however, this association was not statistically significant (OR = 0.64, 95% CI: 0.38~1.09), as did employment (OR = 0.57, 95% CI: 0.36~0.89). Smokers showed a 1.94-fold higher risk of depression compared to non-smokers (OR = 1.94, 95% CI: 1.11~3.38). Alcohol consumption was not included in the final models because it was not significantly associated with depression in univariable analyses and did not alter the associations between glycemic indicators and depression when tested in preliminary models.

## DISCUSSION

This study was conducted to examine changes in depression levels according to glycemic control indices among patients with T2DM. Unlike previous studies that focused primarily on HbA1c, this study highlights the differential associations between depression and glycemic indices reflecting distinct temporal windows of glycemic control.

Regarding general characteristics, the proportion of middle-aged participants was higher than that of older adults, and depression levels were elevated particularly among younger individuals within the depressed group. This finding is consistent with prior research suggesting that the prevalence of T2DM among relatively younger populations is increasing, possibly due to Westernized lifestyles and reduced physical activity [14,15]. However, rather than reiterating demographic characteristics, the present discussion focuses on how these characteristics interact with glycemic control and depressive symptoms. Therefore, nursing interventions are needed to identify and address risk factors such as overweight, obesity, family history of diabetes, and comorbid chronic diseases (e.g., hypertension, dyslipidemia) [4,9] to prevent progression to prediabetes and beyond. Moreover, as there remains insufficient evidence on the clinical course and pharmacological management of younger T2DM patients [4], nursing interventions should also focus on preventing complications and promoting lifestyle modifications tailored to this group.

The prevalence of aerobic physical activity among participants was relatively low (36.3%), and 73.9% were overweight or obese, with only 23.6% maintaining a normal BMI. This is consistent with previous research reporting a mean BMI of 24.2 kg/m^2^ among diabetic patients [5], underscoring that adherence to exercise regimens important to prevent diabetes remains insufficient. Smoking prevalence was 20%, similar to that reported in prior studies (22.6%) [37]. Smoking is known to increase insulin resistance and the risk of cardiovascular complications in diabetes patients, and smoking cessation is an important health-promoting behavior [4]. The finding that smoking was independently associated with higher depression in the final model suggests that lifestyle-related behaviors may exacerbate both metabolic dysregulation and psychological vulnerability, reinforcing the need for integrated behavioral and mental health interventions. Accordingly, nursing assessments should consider lifestyle and metabolic characteristics as contextual factors when evaluating depressive symptoms, rather than as isolated targets of intervention.

In this study, 19.4% of patients with T2DM were classified with high depression levels, exceeding proportions reported in earlier studies [29]. This finding underscores depression as a prevalent and clinically meaningful comorbidity in T2DM, warranting routine screening and timely intervention within diabetes care settings.

The most notable finding of this study is the contrasting associations between depression and fructosamine versus HbA1c. After adjusting for confounding variables, the risk of depression was significantly higher in the normal fructosamine group but significantly lower in the normal HbA1c group. These results suggest that short- to intermediate-term glycemic control and long-term glycemic control may relate differently to depressive symptoms.

Lower fructosamine levels, reflecting recent glycemic control over the past 2~3 weeks [38], may reflect periods of intensified glycemic management, such as dietary restriction or treatment adjustment [8], during which psychological burden may be heightened. In contrast, lower HbA1c levels, reflecting more stable long-term glycemic control, may represent successful adaptation to diabetes self-management [39], thereby contributing to lower levels of depression [30]. Previous studies have suggested that strict glycemic control and early treatment phases may impose a substantial self-management burden on patients, which could influence emotional well-being [8,10]. However, the present findings indicate that psychological factors should be considered as part of the broader context of glycemic management, particularly when interpreting glycemic indices that reflect different temporal windows.

FBG was not significantly associated with depression, suggesting that single-point or short-term glucose measurements may be insufficient to capture the psychosocial dimensions of glycemic control. These findings emphasize that the temporal characteristics of glycemic indices should be considered when interpreting their associations with mental health outcomes.

Although prior studies have reported positive associations between higher HbA1c levels and depression [25,31], evidence regarding fructosamine remains limited. The inverse association observed in this study highlights fructosamine as a potentially sensitive marker of short-term psychosocial stress related to diabetes management. While renal function and other metabolic conditions may influence fructosamine levels, the present study could not determine whether such factors specifically contributed to the higher depression risk observed in the normal fructosamine group; therefore, this explanation should be interpreted cautiously and warrants further investigation [40].

From a clinical and nursing perspective, these findings have important implications. Rather than relying solely on HbA1c, healthcare providers should consider incorporating intermediate-term glycemic indicators, such as fructosamine, when assessing patients’ psychological well-being. Given the high prevalence of depression observed in this study, routine mental health screening, such as the use of the PHQ-9, should be integrated into regular diabetes care to facilitate early identification of depressive symptoms.

Patients who achieve rapid short-term glycemic improvement may require additional emotional support to cope with the psychological burden of intensified self-management. Interpreting glycemic indices within their temporal context, in conjunction with systematic depression assessment, may enable nurses to identify patients at heightened psychological risk during specific phases of diabetes management. Tailored interventions addressing both metabolic targets and mental health may therefore enhance treatment adherence and long-term outcomes in patients with T2DM [41].

In summary, this study contributes to the literature by demonstrating that glycemic indices reflecting different time frames show distinct relationships with depression. These findings support a more nuanced approach to diabetes care, integrating metabolic monitoring with psychological assessment, and provide a foundation for developing targeted nursing interventions for patients at risk of depression.

## CONCLUSION

This descriptive study analyzed data from the KNHANES to investigate the effects of glycemic control indices on depression among Korean patients with T2DM. The findings revealed that patients with higher depression levels had higher HbA1c and lower fructosamine compared with those with lower depression levels. Specifically, depression was differentially associated with glycemic indices reflecting distinct time frames of glycemic control. Factors influencing depression included age, smoking status, body weight, and waist circumference. In particular, patients with higher depression levels were more likely to belong to the poor glycemic control group based on long-term HbA1c levels (OR = 0.22), whereas they were more likely to fall into the well-controlled group based on intermediate-term fructosamine levels (OR = 6.77).

This study has several limitations. First, due to the cross-sectional design, causal relationships between glycemic indices and depression could not be established. Second, factors such as dietary habits, individual circumstances, and emotional states, which may influence depression, were not assessed. Third, comorbid blood disorders, renal disease, or hepatic disease that could affect HbA1c and fructosamine levels were not evaluated.

Despite these limitations, this study is meaningful in that it examined the associations of depression with not only HbA1c and FBG but also fructosamine, an intermediate-term glycemic marker. The contrasting associations observed between fructosamine and HbA1c suggest that short- to intermediate-term and long-term glycemic control may relate differently to depressive symptoms in patients with T2DM. In conclusion, higher depression levels were significantly associated with higher HbA1c and lower fructosamine, whereas no significant association was found with FBG. These findings indicate that the temporal characteristics of glycemic indices should be considered when interpreting their relationships with depression. Future longitudinal studies are needed to clarify the causal relationships between glycemic indices and depression in patients with T2DM.

## Figures and Tables

**Table 1.** Characteristics of the Study Population (N = 845)

Variables	Categories		Total	Non-depressed (n = 681, 80.6%)	Depressed (n = 164, 19.4%)	t, F or χ^2^* (p)*
General characteristics	Age (years)	30~49	105 (12.4)	71 (15.8)	34 (29.8)	17.60 (.002)
		50~69	467 (55.3)	381 (59.3)	86 (50.6)	
		≥ 70	273 (32.3)	229 (24.9)	44 (19.7)	
	Sex	Men	435 (51.5)	365 (58.3)	70 (54.6)	0.74 (.467)
		Women	410 (48.5)	316 (41.7)	94 (45.4)	
	Education level	Elementary school or less	270 (32.0)	215 (25.8)	55 (25.1)	3.01 (.508)
		Middle school	140 (16.6)	113 (14.5)	27 (16.7)	
		High school	237 (28.0)	187 (30.5)	50 (35.0)	
		College or higher	198 (23.4)	166 (29.2)	32 (23.3)	
	Living arrangement	Living with others	671 (79.4)	552 (83.2)	119 (75.7)	5.08 (.060)
		Living alone	174 (20.6)	129 (16.8)	45 (24.3)	
	Employment status	Employed	420 (49.7)	355 (57.3)	65 (48.5)	4.17 (.084)
		Not employed	425 (50.3)	326 (42.7)	99 (51.5)	
Health-behavior factors	Current smoking	Yes	169 (20.0)	124 (21.7)	45 (35.3)	13.90 (.004)
		No	676 (80.0)	557 (78.3)	119 (64.5)	
	Binge drinking	Yes	149 (17.6)	120 (21.9)	29 (19.0)	0.69 (.481)
		No	696 (82.4)	561 (78.1)	135 (81.0)	
	Aerobic physical activity^†^	Yes	307 (36.5)	254 (39.4)	53 (36.0)	0.63 (.514)
		No	534 (63.5)	425 (60.6)	109 (64.0)	
Anthropometric factors	BMI (kg/m^2^)^†^	Underweight (< 18.5)	3 (0.4)	3 (0.7)	0 (0)	6.92 (.239)
		Normal weight (18.5~22.9)	199 (24.1)	170 (22.3)	29 (17.6)	
		Overweight (23.0~24.9)	194 (23.5)	162 (25.2)	32 (19.6)	
		Obese (≥ 25.0)	430 (52.0)	333 (51.8)	97 (62.8)	
	Height (cm)		162.91 ± 0.40	163.15 ± 0.43	163.47 ± 0.90	0.32 (.751)
	Weight (kg)		68.87 ± 0.57	68.56 ± 0.58	72.03 ± 1.34	2.49 (.013)
	Waist circumference (cm)		91.17 ± 0.38	90.56 ± 0.43	93.31 ± 0.84	3.05 (.002)
	Systolic blood pressure (mmHg)		125.10 ± 0.72	124.78 ± 0.77	123.27 ± 1.50	–0.96 (.339)
	Diastolic blood pressure (mmHg)		76.14 ± 0.47	76.13 ± 0.48	78.11 ± 1.16	1.60 (.110)
Glycemic indicators	Fasting blood glucose (mg/dL)^†^	< 120	325 (38.5)	252 (37.6)	73 (46.2)	2.69 (.146)
		≥ 120	503 (59.5)	418 (62.4)	85 (53.8)	
	Fructosamine (µmol/L)^†^	< 210	174 (20.6)	130 (19.4)	44 (27.8)	4.99 (.074)
		≥ 210	654 (77.4)	540 (80.6)	114 (72.2)	
	HbA1c (%)^†^	< 6.5	205 (24.3)	158 (23.6)	47 (29.7)	1.55 (.305)
		≥ 6.5	623 (73.7)	512 (76.4)	111 (70.3)	

Data are presented as unweighted n and weighted % or weighted mean and standard errors. *p*-values were calculated using the chi-square test or complex sample general linear model. Binge drinking was defined as consuming 7 or more standard drinks for men and 5 or more for women on a single occasion at least once a month over the past year. BMI = Body mass index; HbA1c = Glycated hemoglobin. ^†^The sample size for each variable may differ because of missing values.

**Table 2.** Multivariable Logistic Regression Analysis of Factors Associated with Depression

Variables	Categories		Model 1			Model 2			Model 3		
			OR	95% CI	*p*	OR	95% CI	*p*	OR	95% CI	*p*
Glycemic indicators	Fasting blood glucose (mg/dL)	< 120	1.25	0.84~1.86	.269	1.44	0.95~2.17	.083	1.47	0.97~2.23	.067
		≥ 120	1.00			1.00			1.00		
	Fructosamine (µmol/L)	< 210	7.15	1.99~25.68	.003	6.65	1.83~24.15	.004	6.77	1.79~25.56	.005
		≥ 210	1.00			1.00			1.00		
	HbA1c (%)	< 6.5	0.20	0.06~0.69	.011	0.22	0.06~0.76	.017	0.22	0.06~0.79	.021
		≥ 6.5	1.00			1.00			1.00		
General characteristics	Age (years)	30~49				3.75	2.08~6.76	<.001	3.07	1.58~5.99	.001
		50~69				1.484	0.88~2.52	.142	1.30	0.75~2.28	.350
		≥ 70				1.00			1.00		
	Living arrangement	Living with others				0.60	0.37~0.98	.042	0.64	0.38~1.09	.099
		Living alone				1.00			1.00		
	Employment status	Employed				0.60	0.38~0.94	.024	0.57	0.36~0.89	.014
		Not employed				1.00			1.00		
Health behavior factors	Current smoking	Yes							1.94	1.11~3.38	.020
		No							1.00		
			R^2^ = .02			R^2^ = .08			R^2^ = .10		
			F = 3.39			F = 5.12			F = 5.27		
			*p* = .019			*p* < .001			*p* < .001		

Data were analyzed using complex sample multivariable logistic regression. Reference group: Non-depressed. OR = Odds ratio; CI = Confidence interval; HbA1c = Glycated hemoglobin.

## Data Availability

This study used publicly available data from the KNHANES, which are accessible through the KDCA data portal (https://knhanes.kdca.go.kr).
